# HIV–TB Coinfection: Current Therapeutic Approaches and Drug Interactions

**DOI:** 10.3390/v16030321

**Published:** 2024-02-21

**Authors:** Inesa Navasardyan, Rita Miwalian, Aelita Petrosyan, Stephanie Yeganyan, Vishwanath Venketaraman

**Affiliations:** College of Osteopathic Medicine of the Pacific, Western University of Health Sciences, Pomona, CA 91766, USA; inesa.navasardyan@westernu.edu (I.N.); rita.miwalian@westernu.edu (R.M.); aelita.petrosyan@westernu.edu (A.P.); stephanie.yeganyan@westernu.edu (S.Y.)

**Keywords:** HIV, tuberculosis, mycobacterium tuberculosis, antiretroviral therapy, drug–drug interactions

## Abstract

The co-occurrence of human immunodeficiency virus (HIV) and tuberculosis (TB) infection poses a significant global health challenge. Treatment of HIV and TB co-infection often necessitates combination therapy involving antiretroviral therapy (ART) for HIV and anti-TB medications, which introduces the potential for drug–drug interactions (DDIs). These interactions can significantly impact treatment outcomes, the efficacy of treatment, safety, and overall patient well-being. This review aims to provide a comprehensive analysis of the DDIs between anti-HIV and anti-TB drugs as well as potential adverse effects resulting from the concomitant use of these medications. Furthermore, such findings may be used to develop personalized therapeutic strategies, dose adjustments, or alternative drug choices to minimize the risk of adverse outcomes and ensure the effective management of HIV and TB co-infection.

## 1. Introduction

Human immunodeficiency virus (HIV) continues to be a significant global health challenge with approximately 39 million people living with HIV worldwide in 2022, with the majority (25.6 million) in the WHO African Region [1]. In the same year, 630,000 people died from HIV-related causes, and 1.3 million people acquired HIV [1]. HIV is further classified into two main types: HIV-1 and HIV-2. HIV-1, which was identified initially, is widespread globally, whereas HIV-2 is less aggressive and predominantly found in West Africa [1]. HIV and its progression to acquired immunodeficiency syndrome (AIDS) remains a global health issue that requires concerted efforts in education, prevention, and treatment [1]. HIV is an enveloped retrovirus with viral glycoproteins (Gp120 and Gp41) necessary for viral attachment and entry into host cells [2]. HIV carries its genetic information in the form of two identical positive-sense single-stranded RNA molecules enclosed by a capsid to protect the viral RNA and its associated enzymes essential for viral replication [3]. HIV primarily targets CD4+ T lymphocytes through its interaction between viral glycoproteins and CD4 surface receptors, assisted by the co-receptors CCR5 or CXCR4, resulting in the fusion of the viral envelope with the host cell membrane and, consequently, the entry of viral RNA into the host cell [4]. Once inside the host cell, viral RNA undergoes reverse transcription into DNA, which is then able to integrate into the host genome to produce new viral particles [5]. The rapid replication of HIV within the host cell leads to the subsequent destruction of CD4+ T cells and, thus, a compromised immune system and an inability to mount an effective immune response against various opportunistic infections [6]. When the CD4+ T cell count drops below 200 cells/mm^3^, HIV is said to have progressed to AIDS. Antiretroviral therapy (ART) continues to be the standard treatment for HIV/AIDS, often consisting of various classes of ART drugs used in combination to suppress viral activity, enhance immune activity, and prevent disease progression [7]. A standard HIV regimen includes a combination of three anti-HIV medications from a minimum of two drug classes. ART is not curative; however, it has been shown to reduce HIV transmission and prolong the lives of those living with HIV [7].

Mycobacterium tuberculosis (*Mtb*) is the causative agent of tuberculosis (TB). *Mtb* is an acid-fast bacterium with a characteristic cell wall of *Mtb* composed of mycolic acid, which contributes to its resistance to antibiotics and the body’s immune response [8]. *Mtb* is a slow-growing bacterium with a generation time of approximately 18–24 h, contributing to the chronic nature of *Mtb* infection. Upon initial infection with *Mtb*, alveolar macrophages will engulf the bacteria in an effort to control the infection and prevent the dissemination of the bacterium to other parts of the body [9]. *Mtb* is a facultative intracellular pathogen capable of living and replicating in macrophages. However, chemokine expression by macrophages triggers the formation of tumor necrosis factor-alpha (TNF-α), a crucial regulator of granuloma formation that controls *Mtb* infection [10]. The majority of individuals infected with *Mtb* develop latent TB infection (LTBI), in which the bacterium remains dormant in host immune cells for years before progressing to active TB infection [11]. Thus, in latent infections, *Mtb* remains dormant inside macrophages, limiting its ability to disseminate to other parts of the body. 

Those with LTBI may develop active TB infection if the immune system becomes weakened, such as in the case of HIV/AIDS [11]. In individuals with HIV/AIDS, the immune system is compromised, making it more difficult for the body to keep Mtb infection controlled [11]. As a result, the latent infection may become active, leading to the development of TB disease. In the United States, although TB-HIV co-infection rates have decreased over the years due to improved HIV management, it still affects a notable portion of the population. Worldwide, the prevalence of TB–HIV co-infection remains substantial, particularly in regions with a high HIV prevalence and limited access to healthcare [11]. The annual risk of developing active TB disease through the reactivation of latent infection in individuals with untreated HIV is estimated to be around 3–16% [12]. The prognosis for individuals with HIV–TB co-infection depends on various factors, including the stage of both diseases at the time of diagnosis, access to medical care, and adherence to treatment [12]. With a timely diagnosis and appropriate management, one’s prognosis can be significantly improved, but without proper intervention, the combination of TB and HIV can lead to severe complications and increased mortality rates. Therefore, comprehensive strategies focusing on prevention, early detection, and integrated treatment approaches are crucial in addressing the challenges posed by HIV–TB co-infection. However, drug–drug interactions (DDIs) must be considered in the treatment of individuals with HIV and TB co-infection.

## 2. Altered CD4+ T Cell Response in HIV–TB Coinfection

The altered host immune response in individuals with HIV significantly increases their susceptibility to infection with *Mtb* [13]. In immunocompetent individuals, various immune cells work together to identify and target pathogens, such as *Mtb*. CD4+ T lymphocytes are one of these immune cells, playing a critical role in coordinating the immune response against infection. In immunocompromised individuals with HIV, however, the ultimate destruction and reduction in CD4+ T cells weakens the ability of the host immune system to respond effectively to infection with *Mtb*, predisposing individuals with HIV to concurrent TB [13]. 

**Figure 1:**
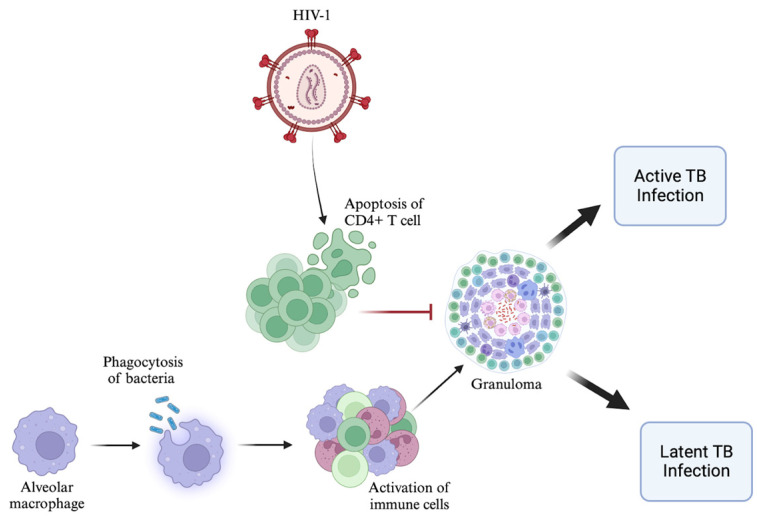
Impact of HIV-induced immunosuppression on host CD4+ T cell response to TB infection. In immunocompetent individuals infected with *Mtb*, CD4+ T lymphocytes along with other immune cells work synergistically to combat infection. CD4+ T cells recognize specific *Mtb* antigens presented by APCs, leading to activation of macrophages and formation of granulomas, which help keep the infection latent within host cells. HIV-induced immunosuppression results in a drastic reduction in functional CD4+ T cells. Impaired recognition of *Mtb* antigens weakens the immune response. Within granulomas, HIV leads to CD4+ T cell apoptosis, causing granuloma disorganization and instability. The compromised immune response in HIV-infected individuals allows *Mtb* to evade immune surveillance, leading to active TB infection.

The initial step in the host immune response against infection is recognition of the invading pathogen. CD4+ T cells play a pivotal role in recognizing specific antigenic peptides associated with *Mtb* presented alongside major histocompatibility complex (MHC) class II molecules on the surface of antigen presenting cells (APCs). With reduced levels of functional CD4+ T cells in individuals with HIV, the immune system is unable to identify and target TB effectively, allowing *Mtb* to persist and multiply in the body. CD4+ T cells are also important for activating other immune cells, such as macrophages, which are primarily responsible for engulfing and digesting pathogens like *Mtb* [14]. When CD4+ T cell numbers are reduced, there is a subsequent reduction in the number of activated macrophages and, thus, less effective clearing of *Mtb*, leading to the persistence of the infection. CD4+ T cells alongside macrophages also play a pivotal role in controlling *Mtb* infection through the formation of granulomas in an effort to contain the bacteria [14]. The hallmark feature of TB is the presence of a caseating granuloma, characterized by epithelioid macrophages surrounding a necrotic core, encased by a layer of both B and T lymphocytes [15]. The formation of granulomas allows some individuals to harbor latent TB infection, wherein the bacteria is present within host macrophages but is not causing active disease [15]. In HIV-infected individuals, the virus enters granulomas, causing apoptosis and the depletion of functional CD4+ T cells, leading to granuloma disorganization and instability, allowing *Mtb* to spread more easily within the body [16]. HIV-induced immunosuppression weakens the body’s control over latent TB, leading to the reactivation of the infection and transformation into active and contagious TB disease (Figure 1).

The altered host immune response in HIV patients is multifaceted, characterized by the depletion of CD4+ T cells, compromising the ability of host immune cells to recognize, respond to, and control TB infection effectively. This weakened immune response allows *Mtb* to evade the immune system’s defenses, leading to increased susceptibility to TB infection and higher rates of TB-related morbidity and mortality in HIV-infected individuals. HIV and TB co-infection presents a challenging public health issue, especially in regions in which both diseases are prevalent. Thus, further advancements in drugs targeting both HIV and TB are crucial in reducing transmission and improving the quality of life of affected individuals.

## 3. HIV: Current Therapeutic Approaches

The development of ART has significantly enhanced the life expectancy of individuals afflicted with HIV. It is imperative that all HIV patients, regardless of the stage of their condition, rigorously adhere to their prescribed medications throughout their lifetime to mitigate mortality and morbidity as well as reduce the likelihood of viral transmission to others. The process of drug selection in HIV treatment and the combination of various antiretroviral agents is tailored to the specific needs of individual patients. The landscape of HIV drug development is continually evolving with the development of drugs targeting one of the pivotal stages in viral replication, including entry, reverse transcription, integration, replication, assembly, and ultimately budding and maturation [17]. The identification and conceptualization of these processes form the basis for the development of the multifaceted antiretroviral therapies currently available in the market.

Nucleoside/nucleotide reverse transcriptase inhibitors (NRTIs) constitute a class of ART used in the treatment of both HIV-1 and HIV-2. This category of drugs includes abacavir sulfate, emtricitabine, lamivudine, tenofovir alafenamide, tenofovir disoproxil fumarate, and zidovudine. NRTIs function as prodrugs that require phosphorylation in order to transform into their active metabolite state. The active forms, known as 2′, 3′-dideoxynucleoside 5′-triphosphates (ddNTPs) or 2′, 3′-dideoxynucleoside 5′-diphosphates (ddNDPs), inhibit the HIV-1 reverse transcriptase (HIV-RT) enzyme via competition with natural nucleosides [18]. Consequently, the incorporation of ddNTPs/ddNDPs results in the premature termination of viral RNA transcription [18]. Despite their efficacy as a primary line of defense against HIV, NRTIs harbor the potential for mitochondrial toxicity due to the interference of NRTIs with polymerase γ, the enzyme responsible for replicating mitochondrial DNA [19]. Consequently, NRTIs interfere with mitochondrial energy production, enzyme inhibition, and electron transport uncoupling. The dysfunction of these processes precipitates cellular death and, ultimately, impacts tissues and organs [19].

In contrast to NRTIs, non-nucleoside reverse transcriptase inhibitors (NNRTIs) exclusively target HIV-1 [20]. NNRTIs consist of two distinct generations of drugs. First-generation NNRTIs, including Delavirdine and Efavirenz, possess a low genetic barrier, necessitating only a single mutation for resistance to develop [20]. In contrast, second-generation NNRTIs, such as Etravirine and Rilpivirine, display greater resilience against genetic resistance [20]. NNRTIs function as noncompetitive inhibitors of HIV-RT and are generally associated with minimal adverse effects; however, it is noteworthy to mention that Efavirenz and Rilpivirine have been associated with neurological and psychiatric complications [20].

Integrase strand transfer inhibitors (INSTIs) are another class of drugs used to combat both HIV-1 and HIV-2 infections and include drugs such as Raltegravir, Elvitegravir, Dolutegravir, and Bictegravir [21]. Integrase executes its action within a large functional nucleoprotein complex known as an intrasome [21]. HIV integrase is an enzyme that catalyzes the reaction by which viral DNA is incorporated into the host genome, which requires divalent cations as cofactors in order to proceed [21]. INSTIs function as a metal chelating scaffold that is able to attract Mg^2+^ [22]. It contains a halogenated benzene side chain which is associated with viral DNA, and it also contains a linker which links the benzene side chain to the core scaffold [23]. INSTIs selectively bind to the active site of integrase located in the intrasome [24]. In clinical trials and clinical practice, INSTI resistance is uncommon [21]. E92Q is the most common initial mutation that has been observed with failure to respond to Elvitegravir treatment [25].

Retroviral protease inhibitors (PIs) are potent agents active against both HIV-1 and HIV-2 [26]. Within the HIV life cycle, the protease cleaves the Gag and Gag-Pol polyprotein precursor encoded by the HIV-1 virus genome to yield mature and active proteins vital for the progression of viral replication [26]. PIs disrupt this process by blocking the active site of the protease, thereby disrupting viral maturation [26]. In comparison to NNRTIs, PIs have a significantly higher genetic barrier to resistance [27]. However, the long-term use of most PIs may potentially lead to the onset of induced metabolic syndromes consisting of dyslipidemia, insulin resistance, and lipodystrophy/lipoatrophy, adversely affecting the overall health of individuals subjected to extended PI therapy [26].

Chemokine receptor 5 (CCR5) inhibitors, such as Maraviroc, represent a novel class of antiretroviral therapeutics designed to prevent HIV-1 infection of CD4+ T cells via the inhibition of the CCR5 receptor [28]. HIV-1 entry into the host cell is dependent upon the CCR5 receptors; however, CCR5 remains an important therapeutic target due to its indispensability in maintaining normal immune function [29]. In particular, its indispensability is noted in individuals who are homozygous for the Δ32 mutation of CCR5, conferring high resistance to HIV-1 infection [29].

Ibalizumab, a post-attachment inhibitor, is a monoclonal antibody that binds CD4+ T cells to inhibit viral entry [30]. Ibalizumab is commonly used in severely affected HIV-1 individuals with multidrug-resistance to various ARTs [30]. Ibalizumab has demonstrated favorable tolerability in clinical trials with some side effects including diarrhea, nausea, dizziness, fatigue, pyrexia, and rash [31]. Ibalizumab resistance may emerge via the decreased expression of the N-linked glycosylation sites of the V5 loop of the envelope gp120 [32]. 

## 4. TB: Current Therapeutic Approaches

Therapeutics targeting TB infection are multifaceted, encompassing the complete eradication of *Mtb* infection, reduced transmission, prevention of relapse, and deterrence of drug resistance. Therapeutic strategies are dependent upon the distinction between LTBI and active TB infection as well as the presence of drug resistance. First-line therapeutics used in the treatment of TB include Rifampin, Isoniazid, Pyrazinamide, and Ethambutol, often referred to as RIPE therapy [33]. According to the CDC, RIPE protocol consists of an intensive phase lasting 2 months, succeeded by a continuation phase lasting either 4 or 7 months, totaling to a treatment duration of 6 to 9 months [33]. This approach is designed to effectively combat TB while minimizing the risk of relapse and drug resistance. Nevertheless, drug-resistant *Mtb* strains necessitate the use of second-line agents such as injectable aminoglycosides, injectable polypeptides, oral and injectable fluoroquinolones, and Bedaquiline [34].

Rifampin exhibits efficacy against a spectrum of bacteria, including Gram-positive cocci, select Gram-negative organisms, Mycobacteria, and Clostridium difficile [35]. Rifampin exerts its effects by inhibiting microbial DNA-dependent RNA polymerase by binding to the rpoB-encoded β subunit [36]. This interaction interferes with RNA synthesis, effectively blocking the elongation of the RNA transcript when it reaches two to three nucleotides in length [36]. Rifampin is lipid-soluble and can be administered both orally and intravenously [35]. Dose-dependent side effects include orange discoloration of tears, sweat, saliva, urine, and feces, alongside gastrointestinal side effects [35]. Additional dose-independent side effects include hypersensitivity reactions such as urticaria, flu-like symptoms, and renal failure [35]. Resistance to Rifampin treatment is seen in bacterial strains with chromosomal mutations in the rpoB gene encoding the rpoB-encoded β subunit of DNA-dependent RNA polymerase [36].

Isoniazid is a prodrug that enters mycobacterial cells through passive diffusion [37]. Once inside the cell, Isoniazid is activated by the catalase-peroxidase enzyme KatG, resulting in the production of radicals and subsequent disruption of mycolic acid synthesis, a vital component of the *Mtb* cell wall [37]. Moreover, the oxidation of Isoniazid, facilitated in the presence of NADH and InhA, results in the formation of Isoniazid–NADH adducts and the suppression of InhA, an enzyme crucial for synthesizing mycolic acids [38]. Furthermore, Isoniazid induces the depletion of nucleic acid pools and triggers metabolic depression within the cell, leading to the further inhibition of its growth and proliferation [37]. Bacterial strains with selective point mutations in KatG exhibit a reduced ability to activate Isoniazid and thus, are observed to develop resistance to this drug [37].

Pyrazinamide, a nicotinamide analogue, exerts its antimicrobial activity exclusively within an acidic environment (pH 5.5) and exerts sterilizing activity against persistent agents under such acidic conditions [39]. Functioning as a prodrug, Pyrazinamide requires activation through enzymatic conversion to pyrazinoic acid catalyzed by pyrazinamidase, an enzyme encoded by the pncA gene within *Mtb* [39]. Pyrazinamide resistance has been documented in *Mtb* strains lacking pyrazinamidase activity, highlighting the critical role of this enzyme in drug activation [40].

Ethambutol functions as a bacteriostatic agent that interferes with bacterial growth by disrupting the biosynthesis of arabinoglycan, a vital component of the bacterial cell wall [41]. Ethambutol exerts its effects through its inhibition of arabinosyl transferase, an enzyme encoded by the bacterial gene embCAB [41]. Adverse effects associated with Ethambutol include visual impairments such as loss of visual acuity due to optic neuropathy, optic neuritis, and retrobulbar neuritis [42]. Additionally, patients may experience peripheral neuropathy and psychosis [42,43]. Resistance to Ethambutol has been linked to mutations within the embB gene. Notably, this resistance appears to be selective, manifesting through specific amino acid substitutions [44]. 

Multidrug-resistant tuberculosis (MDR-TB) strains of *Mtb* demonstrate resistance to Isoniazid and Rifampin, requiring the use of second-line medications [45]. Extensively drug-resistant tuberculosis (XDR-TB) strains are characterized by resistance to Isoniazid, Rifampin, a fluoroquinolone, and either a second-line injectable medication (amikacin, capreomycin, or kanamycin) or, alternatively, bedaquiline or linezolid [34]. This extensive resistance significantly limits treatment options and those that remain are often less effective, more toxic, and more expensive, making XDR TB exceedingly difficult to manage [34]. Drug-resistant strains are particularly concerning in immunocompromised individuals, such as in HIV-positive individuals. The combination of drug resistance and a compromised immune system enhances the complexity of managing TB in these vulnerable populations, emphasizing the need for novel approaches to address co-infections. Several examples of DDIs within these two medication classes has been provided in the table below (Table 1).

## 5. Current Therapeutic Approaches in HIV–TB Coinfection

**Figure 2:**
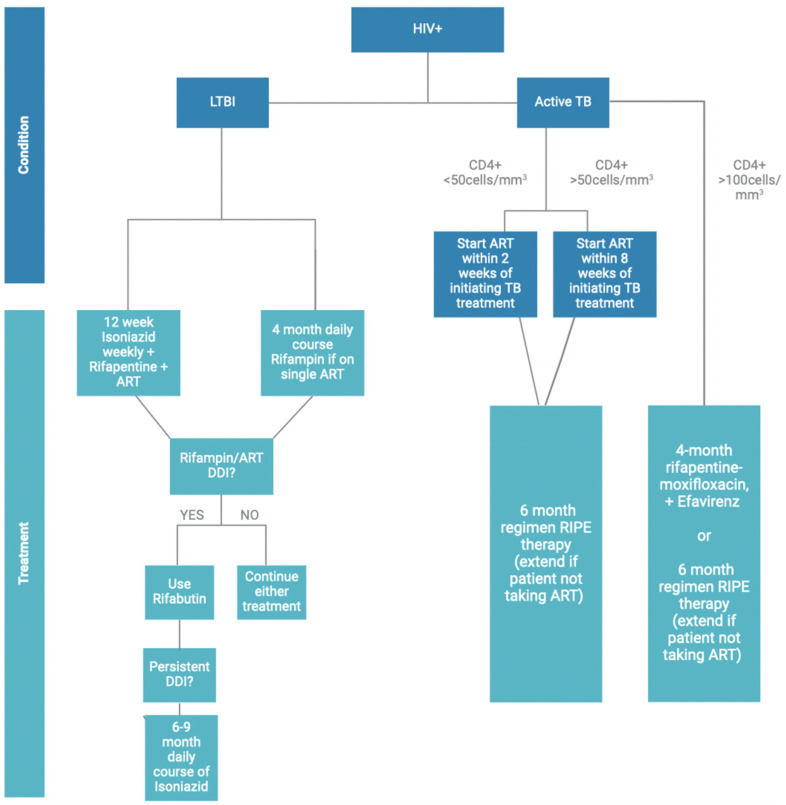
Treatment Regimen for Individuals Co-Infected with HIV and LTBI or Active TB. HIV-positive individuals with untreated LTBI can be treated with a 12-week once-weekly regimen of Isoniazid and Rifapentine in combination with ART or four-month daily course of Rifampin if on ART monotherapy. In cases in which there are DDIs with Rifampin, Rifabutin may be used as an alternative. If the individual is using ARTs that significantly interact with Rifapentine or Rifampin, a 6–9-month daily course of Isoniazid is an appropriate substitution. Initiation of ART in HIV-positive patients with active TB infection depends on their respective CD4+ T cell counts. If CD4+ T cell count is less than 50 cells/mm^3^, ART therapy should be initiated within two weeks of initiating TB treatment. Conversely, in patients with a CD4+ T cell count of 50 cells/mm^3^ or higher, ART therapy can be started within eight weeks of initiating TB treatment. These patients may be started on a 6–9-month RIPE regimen. The 4-month regimen of Rifapentine–Moxifloxacin plus an ART regimen or a 6–9-month RIPE regimen is appropriate for patients with a CD4+ T cell count of 100 cells/mm^3^ or higher.

The increased risk of developing TB in HIV-positive individuals necessitates regular testing for both latent and active TB infection [46]. Managing concomitant TB and HIV infections involves a multifaceted approach, including the combination of ARTs and anti-TB medications as well as addressing other concurrent health conditions and preventing immune reconstitution inflammatory syndrome (IRIS) [12]. Given the complexity of this treatment regimen, clinicians must consider the potential cytotoxicity and potential drug–drug interactions (DDIs) among the various therapeutics. Moreover, the duration of treatment is also an important consideration, with a preference for shorter regimens when feasible to enhance patient compliance. The choice of TB preventive treatment for individuals with HIV is contingent upon their specific ART regimen, highlighting the importance of personalized and comprehensive care for individuals co-infected with TB and HIV [47]. This tailored approach optimizes treatment efficacy and minimizes adverse effects, promoting better overall outcomes for these vulnerable populations. A summary of the current treatment options is summarized in Figure 2. 

**Figure 3:**
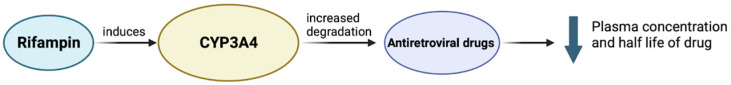
Rifamycin antibiotics are inducers of cytochrome P450 isoenzymes. Pharmacokinetic studies demonstrate the effect of Rifamycin in reducing the half-life and concentration of certain ARTs.

For HIV-positive individuals with untreated LTBI, treatment is critical to prevent the future activation of the infection. Current recommendations from the Center of Disease Control (CDC) describe a 12-week once-weekly regimen of Isoniazid and Rifapentine in combination with ART, provided there are no DDIs [46]. Rifapentine and Rifampin are Rifamycin antibiotics that make up a critical component of TB treatment. They require special consideration when prescribed due to their potential DDIs and their potent cytochrome P450-inducing properties [48]. For example, they are contraindicated in HIV-positive patients taking all protease inhibitors, Doravirine, Rilpivirine, Bictegravir, Cabotegravir, Elvitegravir, Temsavir, and Lenacapavir [47]. Appropriate ARTs include Efavirenz taken once daily or Raltegravir taken twice daily, alongside either abacavir/lamivudine or tenofovir disoproxil fumarate/emtricitabine [47]. However, Rifampin and Rifapentine significantly decrease the concentration of these drugs and would warrant increasing the concentration of the ARTs, which may increase the risk of drug related adverse effects [47]. Pharmacokinetic studies demonstrate the effect of Rifamycin in reducing the concentration of Efavirenz as well [49]. Thus, treatment regimens must be modified to ensure optimal doses for effective patient management. Current guidelines suggest co-administering Rifamycin with Efavirenz at a dose of 600 mg daily; however, some sources recommend increasing the dose to 800 mg daily for individuals weighing more than 60 kg [50]. This adjustment in dosage is essential to maintain adequate drug levels to ensure efficacy of both TB and HIV treatments while minimizing the risk of treatment failure and drug resistance (Figure 3). Dolutegravir 50 mg once daily may also be used as a first line ART but is not recommended in patients with integrase strand transfer inhibitor resistance [47]. Although not highlighted as standard of care, nucleoside/nucleotide reverse transcriptase inhibitors, Enfuvirtide, and Ibalizumab were not found to have significant DDIs with rifamycin, which may be implemented if deemed appropriate by the clinician [47]. Alternatively, a 4-month daily course of Rifampin may be prescribed to individuals on ART monotherapy, such as Efavirenz and should not be given to individuals on a combination of ARTs [46,47]. In cases where an ART with DDIs with Rifampin is already established, Rifabutin may be used as an alternative. Rifabutin is another drug in the Rifamycin family that is effective in treating *Mtb* [51]. In a study of patients enrolled with a Rifampin-related adverse effect, 80% were treated with Rifabutin successfully demonstrating its use in patients who have Rifampin-related adverse effects or DDIs [51]. Another alternative in individual using ARTs that significantly interact with Rifampin, a 6–9-month daily course of Isoniazid is also an appropriate substitution, and any ART regimen may be used in conjunction with this treatment [46]. Figure 3 Recently, there has been growing popularity for even shorter drug courses over traditional courses since they are effective and have greater completion rates [52]. In the BRIEF TB/AF279 clinical trial, researchers found that a 1-month regimen of Rifapentine and Isoniazid was comparable in efficacy to 9 months of Isoniazid in preventing TB infection in HIV-positive patients [53]. This ultra-short regimen demonstrated a greater completion rate and is now recommend by WHO [53,54].

Initiation of ART in HIV-positive patients with active TB infection depends on their respective CD4+ T cell counts [47]. If CD4+ T cell count is less than 50 cells/mm^3^ ART therapy should be initiated within two weeks of initiating TB treatment [47]. Conversely, in patients with a CD4+ T cell count of 50 cells/mm^3^ or higher, ART therapy can be started within eight weeks of initiating TB treatment [48]. There are currently two mainstay treatment options for individuals with active TB and HIV infection. The first option is a six-to-nine-month regimen consisting of an intensive phase with Rifampin, Isoniazid, Pyrazinamide, and Ethambutol for two months, followed by a continuation phase with Rifampin and Isoniazid for 4 months, as recommended by the CDC [55]. In rare cases in which the patient does not receive ART, the duration of this treatment should be extended [55]. The second option consists of a 4-month regimen of Rifapentine–Moxifloxacin, suitable for patients with a CD4+ T cell count of 100 cells/microliter and an ART regimen such as Efavirenz [46]. This treatment involves an 8-week intensive phase comprising Rifapentine, Moxifloxacin, Isoniazid, and Pyrazinamide, followed by a 9-week continuation phase with the same drugs except Pyrazinamide. 

## 6. Potential Therapeutic Strategies for HIV–TB Coinfection

Despite current therapeutic strategies, the risk of mortality among HIV-positive patients is markedly increased by concurrent infection with *Mtb* [56]. Many of the limitations of the available therapeutic options are due to drug resistance, adverse reactions, and the synergistic behavior between *Mtb* and HIV with each pathogen potentiating the other’s pathogenesis [57]. Thus, novel therapeutic strategies are necessary in preventing and combating co-infection to improve survival rates and overall patient well-being.

**Figure 4:**
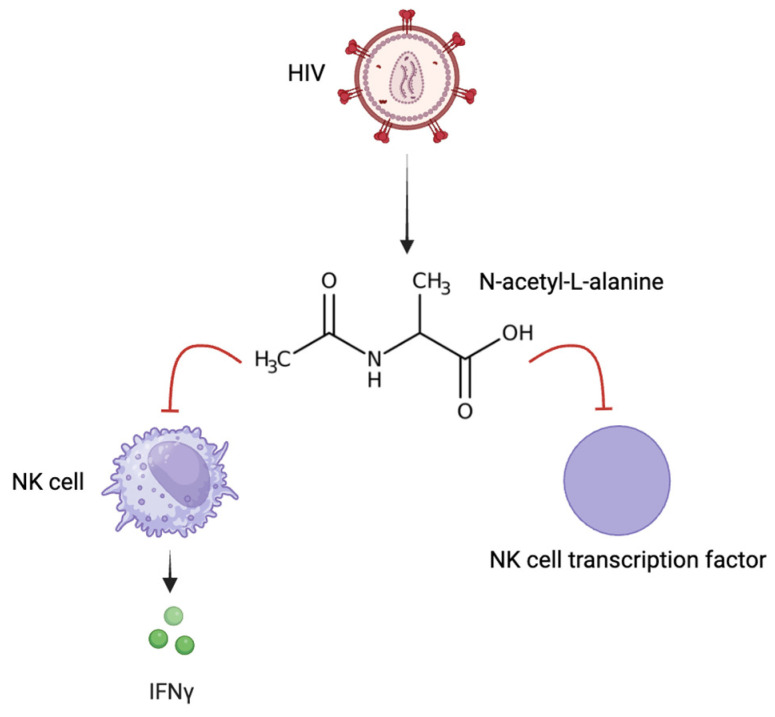
Impact of HIV on cell-mediated immune response. HIV-infected individuals present with increased levels of N-acetyl-alanine, which inhibits natural killer (NK) cells from producing IFN-γ, a proinflammatory cytokine, in addition to NK cell transcription factors for antimicrobial properties against *Mtb*.

One proposed potential therapeutic strategy includes the use of dual-targeted anti-HIV/anti-TB heterodimers [57]. Of five synthesized compounds, four of them were dimers of anti-TB and anti-HIV agents and therefore inhibited both pathogens. One compound was a heterodimer of one anti-TB and two anti-HIV reverse transcriptase inhibitors. All compounds were found to be successful in inhibiting both infections in vivo and ex vitro [57]. Additionally, there was no significant cytotoxicity for any of the five heterodimers. Another study targeted HIV’s inhibition of natural killer(NK) cell-mediated immunity in response to *Mtb* infection [58]. HIV-infected individuals display increased levels of the metabolite *N*-acetyl-l-alanine (ALA), which inhibits the proinflammatory cytokine IFN-γ. IFN-γ is produced by NK cells of latent *Mtb*-infected individuals. ALA diminishes NK cell glycolysis and further represses the expression of transcription factors with antimicrobial properties of NK cells against *Mtb* [58]. The inhibition of IFN-γ due to elevated ALA levels from HIV infection demonstrates the synergistic behavior between HIV and *Mtb* in coinfection [58]. Although treatment with ART has been successful in restoring normal levels of some metabolites, it continues to induce mitochondrial damage, contributing to metabolic dysfunction and decreased energy stores [59]. Further research on the metabolism of HIV–*Mtb* co-infection as metabolite targeting through nutritional therapy in *Mtb*–HIV may be of therapeutic significance [58] (Figure 4).

The targeting of immune cells is another therapeutic strategy that needs further exploration. Both HIV and *Mtb* infect macrophages in the lungs and manipulate endolysosomal components to evade destruction. Cystatin C is an endogenous protease inhibitor that can be internalized by immune cells. A study conducted on the silencing of Cystatin C improves the intracellular killing of *Mtb*. Furthermore, the silencing of Cystatin C leads to increased CD4+ T cell proliferation and increased IFN-γ secretion [60]. Additionally, researchers have found that transcripts representing the classical complement pathway and Fcγ receptor 1 levels increase in subclinical disease of HIV–TB coinfection. Circulating immune complexes increased with the increase in the C1q receptor transcript that binds these complexes. These studies demonstrate the significant need for future research in targeting the genomics of the immune system to slow the progression of co-infection [61].

## 7. Conclusions

Co-infection with HIV and *Mtb* poses a significant risk to individuals due to the presence of drug–drug interactions between anti-HIV and anti-TB therapeutics that may complicate treatment approaches. Moreover, the synergistic nature between the two pathogens further complicates infection. HIV infection targets and inhibits the activity of immune cells, including CD4+ T lymphocytes and macrophages, leading to a vulnerable state due to the inability of the immune system to effectively identify and target *Mtb* invasion. The dual management of HIV and TB infection with the combination of ARTs and anti-TB medications is multifaceted and complex when considering cytotoxicity, drug–drug interactions, and patient compliance. ARTs used to combat HIV infection include NRTIs, NNRTIs, INSTIs, retroviral PIs, CCR5 inhibitors, and Ibalizumab. Although advancements in treatment regimens have proven to be successful in suppressing HIV infection, the potential for adverse side effects hinders its effectiveness. For instance, NRTIs have been shown to cause mitochondrial toxicity reducing energy stores, while NNRTIs are known to induce neurological complications. Additionally, immune resistance to treatment may develop, such as with the use of Ibalizumab. Moreover, treatment regimens for *Mtb* include a 6–9-month course of a combination of antibiotics including the first-line agents Rifampin, Isoniazid, Pyrazinamide, and Ethambutol. Oftentimes, infection with drug-resistant strains of *Mtb* require the use of second-line antibiotics. Rifamycin, another anti-TB antibiotic, upregulates cytochrome P450 enzymes, altering the metabolism of some ARTs used for HIV infection and thus, requires dose adjustments when used in combination. Research has been conducted on potential therapeutic strategies with promising studies on the dual-action use of heterodimers, targeting of metabolites, and transcript silencing with a reduction in cytotoxicity in comparison to current treatment strategies. Nevertheless, there remains an urgent need for further research, particularly on the metabolites of HIV–TB co-infection, as metabolite therapy may provide a promising future for co-infected individuals. 

## Figures and Tables

**Table 1:** Drug–drug interactions (DDIs) between standard anti-HIV and anti-TB therapies. Various drug interactions exist between the standard anti-HIV therapies (vertical column) and RIPE therapy targeting TB (horizontal column).

	Rifampin	Isoniazid	Pyrazinamide	Ethambutol
**Emtricitabine**	No known interactions	No known interactions	No known interactions	No known interactions
**Delavirdine**	No known interactions	No known interactions	No known interactions	No known interactions
**Raltegravir**	Rifampin decreases Raltegravir by increasing hepatic clearance	No known interactions	No known interactions	No known interactions
**Atazanavir**	Contraindicated. Rifampin decreases levels of atazanavir by increasing metabolism and effects CYP3A4 metabolism.	Isoniazid increases the effect of Atazanavir by affecting CYP3A4 metabolism	No known interactions	No known interactions
**Maraviroc**	Rifampin effects CYP3A4 metabolism and decreases effects of Maraviroc through P-glycoprotein (MDR1) efflux transporter	Isoniazid effects CYP3A4 metabolism.	No known interactions	No known interactions

## References

[1] World Health Organization (WHO) HIV and AIDS. https://www.who.int/news-room/fact-sheets/detail/hiv-aids.

[2] Li Y., Liu D., Wang Y., Su W., Liu G., Dong W. (2021). The Importance of Glycans of Viral and Host Proteins in Enveloped Virus Infection. Front. Immunol..

[3] German Advisory Committee Blood (Arbeitskreis Blut), Subgroup ‘Assessment of Pathogens Transmissible by Blood’ (2016). Human Immunodeficiency Virus (HIV). Transfus. Med. Hemother..

[4] Woodham A.W., Skeate J.G., Sanna A.M., Taylor J.R., Da Silva D.M., Cannon P.M., Kast W.M. (2016). Human Immunodeficiency Virus Immune Cell Receptors, Coreceptors, and Cofactors: Implications for Prevention and Treatment. AIDS Patient Care STDS.

[5] Hu W.S., Hughes S.H. (2012). HIV-1 reverse transcription. Cold Spring Harb. Perspect. Med..

[6] Vidya Vijayan K.K., Karthigeyan K.P., Tripathi S.P., Hanna L.E. (2017). Pathophysiology of CD4+ T-Cell Depletion in HIV-1 and HIV-2 Infections. Front. Immunol..

[7] Foka F.E.T., Mufhandu H.T. (2023). Current ARTs, Virologic Failure, and Implications for AIDS Management: A Systematic Review. Viruses.

[8] Holzheimer M., Buter J., Minnaard A.J. (2021). Chemical Synthesis of Cell Wall Constituents of Mycobacterium tuberculosis. Chem. Rev..

[9] Woo M., Wood C., Kwon D., Park K.P., Fejer G., Delorme V. (2018). *Mycobacterium tuberculosis* Infection and Innate Responses in a New Model of Lung Alveolar Macrophages. Front. Immunol..

[10] Domingo-Gonzalez R., Prince O., Cooper A., Khader S.A. (2016). Cytokines and Chemokines in Mycobacterium tuberculosis Infection. Microbiol. Spectr..

[11] Kiazyk S., Ball T.B. (2017). Latent tuberculosis infection: An overview. Can. Commun. Dis. Rep..

[12] Bruchfeld J., Correia-Neves M., Källenius G. (2015). Tuberculosis and HIV Coinfection. Cold Spring Harb. Perspect. Med..

[13] Bares S.H., Swindells S. (2020). Latent Tuberculosis and HIV Infection. Curr. Infect. Dis. Rep..

[14] de Martino M., Lodi L., Galli L., Chiappini E. (2019). Immune Response to *Mycobacterium tuberculosis*: A Narrative Review. Front. Pediatr..

[15] Cronan M.R. (2022). In the Thick of It: Formation of the Tuberculous Granuloma and Its Effects on Host and Therapeutic Responses. Front. Immunol..

[16] Diedrich C.R., O’Hern J., Wilkinson R.J. (2016). HIV-1 and the Mycobacterium tuberculosis granuloma: A systematic review and meta-analysis. Tuberculosis.

[17] Lingappa J.R., Lingappa V.R., Reed J.C. (2021). Addressing Antiretroviral Drug Resistance with Host-Targeting Drugs-First Steps towards Developing a Host-Targeting HIV-1 Assembly Inhibitor. Viruses.

[18] Holec A.D., Mandal S., Prathipati P.K., Destache C.J. (2017). Nucleotide Reverse Transcriptase Inhibitors: A Thorough Review, Present Status and Future Perspective as HIV Therapeutics. Curr. HIV Res..

[19] Kohler J.J., Lewis W. (2007). A brief overview of mechanisms of mitochondrial toxicity from NRTIs. Environ. Mol. Mutagen..

[20] Usach I., Melis V., Peris J.E. (2013). Non-nucleoside reverse transcriptase inhibitors: A review on pharmacokinetics, pharmacodynamics, safety and tolerability. J. Int. AIDS Soc..

[21] Zhao A.V., Crutchley R.D., Guduru R.C., Ton K., Lam T., Min A.C. (2022). A clinical review of HIV integrase strand transfer inhibitors (INSTIs) for the prevention and treatment of HIV-1 infection. Retrovirology.

[22] Barreca M.L., Ferro S., Rao A., De Luca L., Zappalà M., Monforte A.M., Debyser Z., Witvrouw M., Chimirri A. (2005). Pharmacophore-based design of HIV-1 integrase strand-transfer inhibitors. J. Med. Chem..

[23] Smith S.J., Zhao X.Z., Passos D.O., Pye V.E., Cherepanov P., Lyumkis D., Burke T.R., Hughes S.H. (2021). HIV-1 Integrase Inhibitors with Modifications That Affect Their Potencies against Drug Resistant Integrase Mutants. ACS Infect. Dis..

[24] Espeseth A.S., Felock P., Wolfe A., Witmer M., Grobler J., Anthony N., Egbertson M., Melamed J.Y., Young S., Hamill T. (2000). HIV-1 integrase inhibitors that compete with the target DNA substrate define a unique strand transfer conformation for integrase. Proc. Natl. Acad. Sci. USA.

[25] Hurt C.B., Sebastian J., Hicks C.B., Eron J.J. (2014). Resistance to HIV integrase strand transfer inhibitors among clinical specimens in the United States, 2009–2012. Clin. Infect Dis..

[26] Lv Z., Chu Y., Wang Y. (2015). HIV protease inhibitors: A review of molecular selectivity and toxicity. HIV AIDS.

[27] Riddler S.A., Haubrich R., DiRienzo A.G., Peeples L., Powderly W.G., Klingman K.L., Garren K.W., George T., Rooney J.F., Brizz B. (2008). AIDS Clinical Trials Group Study A5142 Team. Class-sparing regimens for initial treatment of HIV-1 infection. N. Engl. J. Med..

[28] Rao P.K. (2009). CCR5 inhibitors: Emerging promising HIV therapeutic strategy. Indian J. Sex. Transm. Dis. AIDS.

[29] Askew D., Su C.A., Barkauskas D.S., Dorand R.D., Myers J., Liou R., Nthale J., Huang A.Y. (2016). Transient Surface CCR5 Expression by Naive CD8+ T Cells within Inflamed Lymph Nodes Is Dependent on High Endothelial Venule Interaction and Augments Th Cell-Dependent Memory Response. J. Immunol..

[30] Emu B., Fessel J., Schrader S., Kumar P., Richmond G., Win S., Weinheimer S., Marsolais C., Lewis S. (2018). Phase 3 Study of Ibalizumab for Multidrug-Resistant HIV-1. N. Engl. J. Med..

[31] Beccari M.V., Mogle B.T., Sidman E.F., Mastro K.A., Asiago-Reddy E., Kufel W.D. (2019). Ibalizumab, a Novel Monoclonal Antibody for the Management of Multidrug-Resistant HIV-1 Infection. Antimicrob. Agents Chemother..

[32] Pace C.S., Fordyce M.W., Franco D., Kao C.Y., Seaman M.S., Ho D.D. (2013). Anti-CD4 monoclonal antibody ibalizumab exhibits breadth and potency against HIV-1, with natural resistance mediated by the loss of a V5 glycan in envelope. J. Acquir. Immune Defic. Syndr..

[33] Centers for Disease Control and Prevention Treatment for TB Disease. https://www.cdc.gov/tb/topic/treatment/tbdisease.htm.

[34] Seung K.J., Keshavjee S., Rich M.L. (2015). Multidrug-Resistant Tuberculosis and Extensively Drug-Resistant Tuberculosis. Cold Spring Harb. Perspect. Med..

[35] Beloor Suresh A., Rosani A., Patel P., Wadhwa R. (2023). Rifampin. StatPearls [Internet].

[36] Koch A., Mizrahi V., Warner D.F. (2014). The impact of drug resistance on Mycobacterium tuberculosis physiology: What can we learn from rifampicin?. Emerg. Microbes Infect..

[37] Timmins G.S., Deretic V. (2006). Mechanisms of action of isoniazid. Mol. Microbiol..

[38] Marrakchi H., Lanéelle M.A., Daffé M. (2014). Mycolic acids: Structures, biosynthesis, and beyond. Chem. Biol..

[39] Zhang Y., Shi W., Zhang W., Mitchison D. (2013). Mechanisms of Pyrazinamide Action and Resistance. Microbiol. Spectr..

[40] Ramirez-Busby S.M., Valafar F. (2015). Systematic review of mutations in pyrazinamidase associated with pyrazinamide resistance in Mycobacterium tuberculosis clinical isolates. Antimicrob. Agents Chemother..

[41] Palomino J.C., Martin A. (2014). Drug Resistance Mechanisms in Mycobacterium tuberculosis. Antibiotics.

[42] Geyer H.L., Herskovitz S., Slamovits T.L., Schaumburg H.H. (2014). Optochiasmatic and peripheral neuropathy due to ethambutol overtreatment. J. Neuroophthalmol..

[43] Behera C., Krishna K., Singh H.R. (2014). Antitubercular drug-induced violent suicide of a hospitalised patient. BMJ Case Rep..

[44] Bakuła Z., Napiórkowska A., Bielecki J., Augustynowicz-Kopeć E., Zwolska Z., Jagielski T. (2013). Mutations in the embB gene and their association with ethambutol resistance in multidrug-resistant Mycobacterium tuberculosis clinical isolates from Poland. Biomed. Res. Int..

[45] Ennassiri W., Jaouhari S., Sabouni R., Cherki W., Charof R., Filali-Maltouf A., Lahlou O. (2018). Analysis of isoniazid and rifampicin resistance in Mycobacterium tuberculosis isolates in Morocco using GenoType^®^ MTBDRplus assay. J. Glob. Antimicrob. Resist..

[46] Centers for Disease Control and Prevention TB Treatment for Persons with HIV. https://www.cdc.gov/tb/topic/treatment/tbhiv.htm.

[47] Panel on Antiretroviral Guidelines for Adults and Adolescents Guidelines for the Use of Antiretroviral Agents in Adults and Adolescents with HIV. Department of Health and Human Services. https://clinicalinfo.hiv.gov/en/guidelines/adult-and-adolescent-arv.

[48] Niemi M., Backman J.T., Fromm M.F., Neuvonen P.J., Kivistö K.T. (2003). Pharmacokinetic interactions with rifampicin: Clinical relevance. Clin. Pharmacokinet..

[49] López-Cortés L.F., Ruiz-Valderas R., Viciana P., Alarcón-González A., Gómez-Mateos J., León-Jimenez E., Sarasanacenta M., López-Pua Y., Pachón J. (2002). Pharmacokinetic interactions between efavirenz and rifampicin in HIV-infected patients with tuberculosis. Clin. Pharmacokinet..

[50] Schutz C., Meintjes G., Almajid F., Wilkinson R.J., Pozniak A. (2010). Clinical management of tuberculosis and HIV-1 co-infection. Eur. Respir. J..

[51] Horne D.J., Spitters C., Narita M. (2011). Experience with rifabutin replacing rifampin in the treatment of tuberculosis. Int. J. Tuberc. Lung Dis..

[52] Ignatius E.H., Swindells S. (2020). Are We There Yet? Short-Course Regimens in TB and HIV: From Prevention to Treatment of Latent to XDR TB. Curr. HIV/AIDS Rep..

[53] Swindells S., Ramchandani R., Gupta A., Benson C.A., Leon-Cruz J., Mwelase N., Jean Juste M.A., Lama J.R., Valencia J., Omoz-Oarhe A. (2019). One Month of Rifapentine plus Isoniazid to Prevent HIV-Related Tuberculosis. N. Engl. J. Med..

[54] (2020). WHO Consolidated Guidelines on Tuberculosis: Tuberculosis Preventive Treatment: Module 1: Prevention [Internet].

[55] Carr W., Kurbatova E., Starks A., Goswami N., Allen L., Winston C. (2022). Interim Guidance: 4-Month Rifapentine-Moxifloxacin Regimen for the Treatment of Drug-Susceptible Pulmonary Tuberculosis—United States, 2022. MMWR Morb. Mortal Wkly. Rep..

[56] Azevedo-Pereira J.M., Pires D., Calado M., Mandal M., Santos-Costa Q., Anes E. (2023). HIV/Mtb Co-Infection: From the Amplification of Disease Pathogenesis to an “Emerging Syndemic”. Microorganisms.

[57] Alexandrova L., Zicari S., Matyugina E., Khandazhinskaya A., Smirnova T., Andreevskaya S., Chernousova L., Vanpouille C., Kochetkov S., Margolis L. (2017). Dual-targeted anti-TB/anti-HIV heterodimers. Antivir. Res..

[58] Yang B., Mukherjee T., Radhakrishnan R., Paidipally P., Ansari D., John S., Vankayalapati R., Tripathi D., Yi G. (2023). HIV-Differentiated Metabolite N-Acetyl-L-Alanine Dysregulates Human Natural Killer Cell Responses to Mycobacterium tuberculosis Infection. Int. J. Mol. Sci..

[59] Herbert C., Luies L., Loots D.T., Williams A.A. (2023). The metabolic consequences of HIV/TB co-infection. BMC Infect. Dis..

[60] Pires D., Calado M., Velez T., Mandal M., Catalão M.J., Neyrolles O., Lugo-Villarino G., Vérollet C., Azevedo-Pereira J.M., Anes E. (2021). Modulation of Cystatin C in Human Macrophages Improves Anti-Mycobacterial Immune Responses to Mycobacterium tuberculosis Infection and Coinfection With HIV. Front. Immunol..

[61] Esmail H., Lai R.P., Lesosky M., Wilkinson K.A., Graham C.M., Horswell S., Coussens A.K., Barry C.E., O’Garra A., Wilkinson R.J. (2018). Complement pathway gene activation and rising circulating immune complexes characterize early disease in HIV-associated tuberculosis. Proc. Natl. Acad. Sci. USA.

